# Cutaneous Larva Migrans (CLM) may not be easy to diagnose: a case report and narrative review

**DOI:** 10.1093/omcr/omae025

**Published:** 2024-04-25

**Authors:** Solafa Osman, Nectaria Tarnari, Areeba Ahsan, Khabab Abbasher Hussien Mohamed Ahmed

**Affiliations:** Assistant Professor of Dermatology, Internal Security Force, Doha, Qatar; Assistant Professor of Dermatology, Internal Security Force, Doha, Qatar; School of Health Sciences, Foundation University, Islamabad, Pakistan; Faculty of Medicine, University of Khartoum, Khartoum, Sudan

**Keywords:** cutaneous larva, Migrance, hookworm, Loeffler’s syndrome, creeping

## Abstract

Background: Cutaneous Larva Migrans (CLM) is one of the most common zoonotic dermatoses in subtropical and tropical regions and some European countries. It is caused by different types of hookworm, such as Ancylostoma braziliense, Ancylostoma caninum*,* and Uncinaria stenocephala. It is usually easy to diagnose, but the atypical presentation may occasionally mimic other dermatoses. Case report: A 32-year-old man presented with an extensive eczematous rash that developed during a recent vacation in Thailand. He didn’t respond to antihistamines and systemic steroids. Finally, he was diagnosed with an atypical presentation of CLM and treated successfully with anthelminthic therapy. Conclusion: The report of an atypical presentation of CLM is crucial to increase awareness among healthcare workers, helping in early diagnosis and reducing potential psychological distress that patients may face.

## INTRODUCTION

CLM is a skin infection caused by hookworms that enter human skin through contact with animal faeces in soil [1]. This creeping eruption, ground itch, or sandworm—all are synonymous with Cutaneous Larva Migrans (CLM), which is one of the most common zoonotic dermatoses in subtropical and tropical regions such as the Caribbean, Brazil, India, and Thailand [1–3]. Also, there are some case reports from European countries such as Germany, England, France, Italy, Spain, and Serbia [4]. It is most common among travellers and tourists [5, 6].

Cutaneous larva migrans (CLM) have a global distribution but are commonly reported in travellers to Africa, South America, Asia, and the Caribbean. Infection often occurs at beaches and sandboxes where free-roaming dogs and cats defecate, and can affect both short-term and long-term travellers [2]. According to population-based studies, the prevalence of cutaneous larva migrans (CLM) can reach up to 8%. However, the prevalence varies significantly between different sexes and age groups [3]. Although cutaneous larva migrans (CLM) are commonly reported in tropical and subtropical regions, cases are now spreading and should be recognized even in urban, non-tropical settings [4]. In semi-arid north-eastern Brazil, the prevalence of cutaneous larva migrans (CLM) ranged from 0.2% to 4.4% in the general population. [5] A study across 30 GeoSentinel sites showed that 2%–3% of 17 000 ill travellers had cutaneous larva migrans (CLM). In France, the prevalence of CLM was estimated at 1-3% in ill travellers returning from endemic countries [6].

It is usually easy to diagnose, but occasionally, atypical presentation becomes very strange and mimics other dermatoses, leading to a delay in the diagnosis and subsequently causing financial and psychological distress to the patients [5].

Clinical presentation can get complicated by bacterial infection due to intense itching and scratching. Pain may occur in papulovesicular lesions, while systemic symptoms, such as peripheral eosinophilia, migratory pulmonary infiltrates, and increased immunoglobulin E levels, are rare [7]. Differential diagnoses include scabies, erythema chronicum migrans, larva currens, epidermal dermatophyte infection and phytophotodermatitis [8]. Cutaneous Larva Migrans is diagnosed based on clinical presentation and exposure history. However, there may be variations in diagnostic accuracy due to the reliance on clinical judgment [9].

Therefore, the report of atypical presentations is essential to raise awareness among health workers [7].

## CASE PRESENTATION

**Figure 1:**
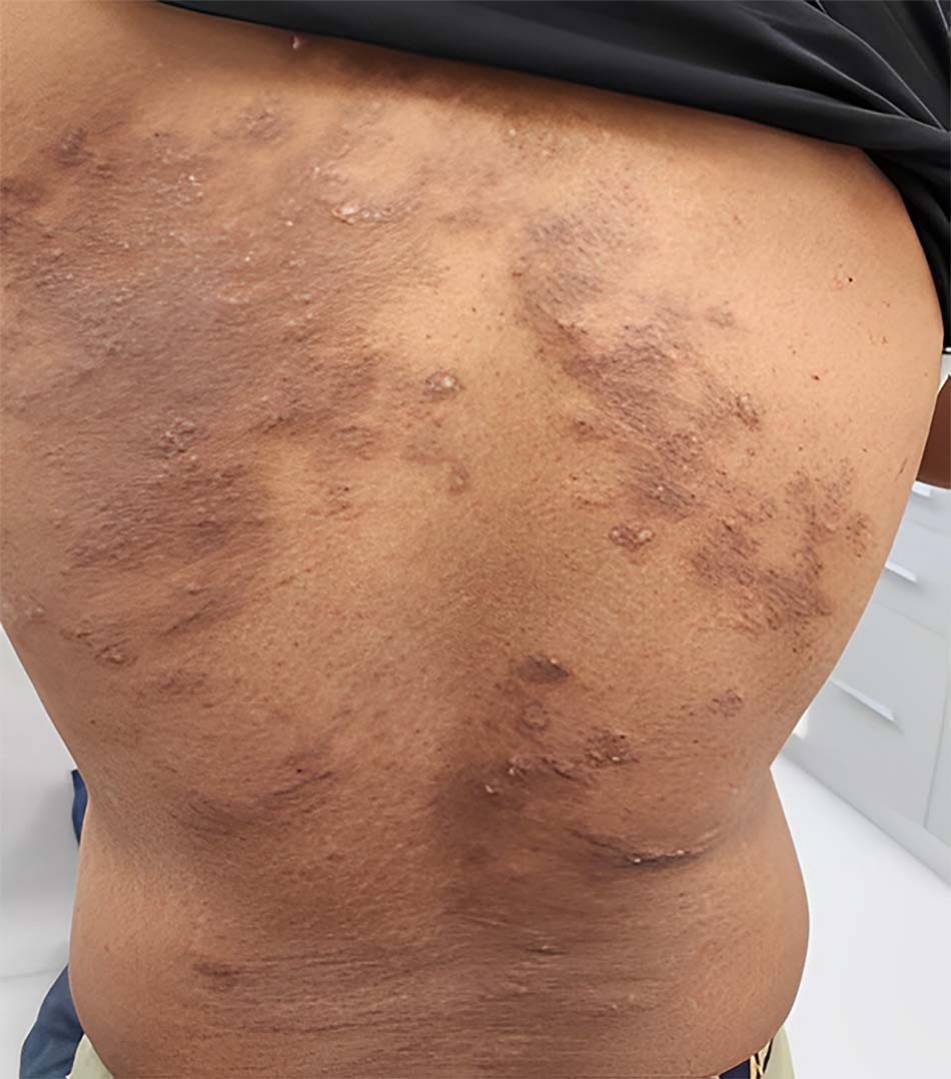
Hyperpigmented and erythematous patches with an inflammatory border, and atypical serpiginous patterns in some areas.

**Figure 2:**
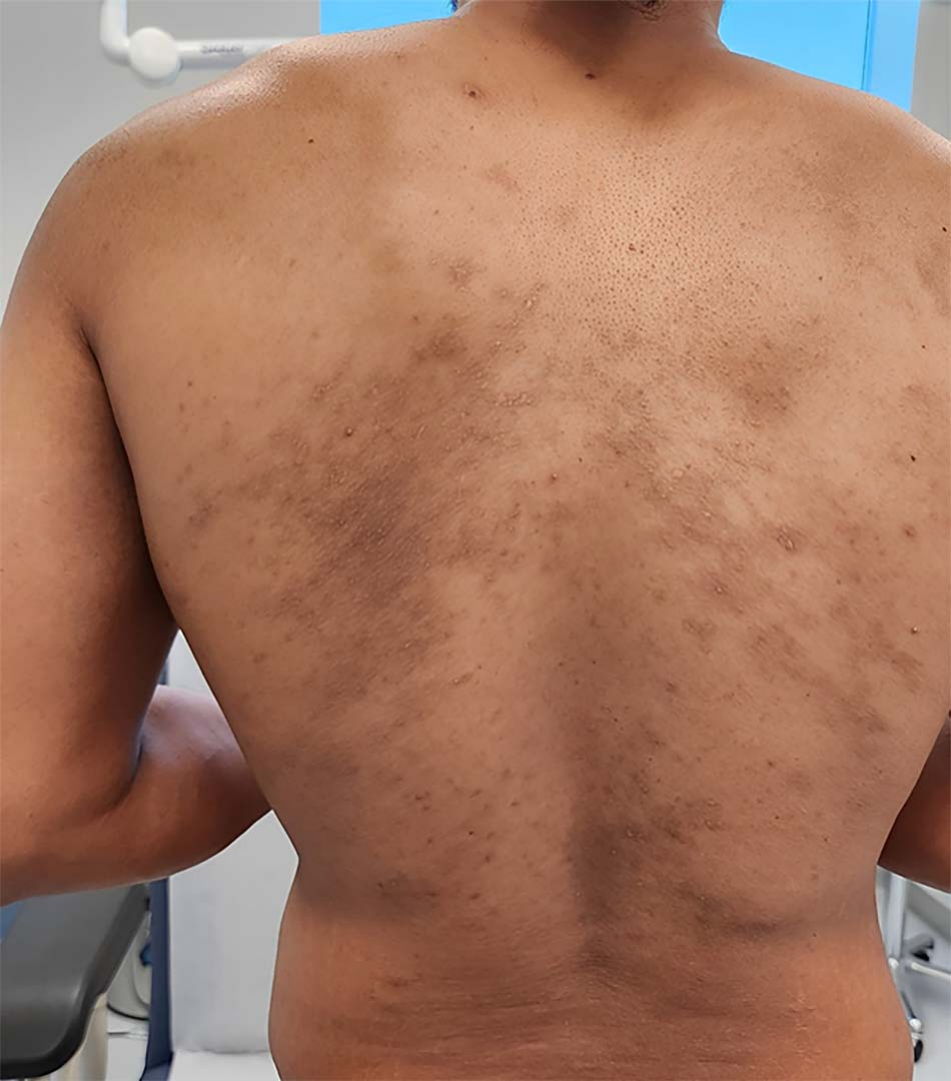
The lesions and itching resolved completely within two weeks of treatment.

After travelling to Thailand, a 32-year-old Sudanese male presented with a one-month history of skin eruption with severe itching on his back. He developed the ailment after lying for about a minute on a rock, and then he began to feel a stinging on his back, which increased gradually and progressed to severe itching at night, preventing him from sleeping. The patient rated their itch intensity as 8 out of 10 on a numerical rating scale (NRS) where 0 represents no itch and 10 represents the worst possible itch. He noticed pimples in the same region a day later. He went to the doctor, who prescribed prednisolone 15 mg, antihistamine tabs, and calamine lotion. He felt mild relief, but there was still tremendous itching, and new lesions emerged. He consulted another doctor, who told him to continue taking the same medication. Then, he visited our clinic a month after the onset of his illness. The examination of his back revealed hyperpigmented patches, erythematous papules, and plaques, and in certain places, atypical serpiginous patterns were observed (Fig. 1). The patient had no significant medical, dermatological, or drug history. Investigations were requested to inform of CBC, ESR, and IgE. At the same time, as an immediate action, the dosage of prednisolone was increased to 40 mg, along with antihistamines and topical emollients. The patient returned after one week with remarkable improvement in the itching, but new lesions still appeared. The investigation result showed an increase in TWBcs, Eosinophils, and IgE levels. A comprehensive clinical examination that focused on dermatoscopic features was conducted and the characteristic serpiginous tracks left by larval migration beneath the skin were identified. The presence of these itchy hyperpigmented erythematous patches and serpiginous tracks on the skin, CBC findings, and raised IgE levels, along with the patient’s recent travel history to Thailand, a tropical country led to the diagnosis of CLM. We ruled out other infestations like scabies or onchocerciasis. Scabies causes discreet lesions in different areas of the body such as interdigital spaces and flexural surfaces, while dermatophyte infections like tinea corporis cause red, ring-shaped rashes but lack the characteristic serpiginous tracks of CLM. After ruling out these infestations, the diagnosis of Cutaneous Larva Migrans (CLM) with a severe subsequent eczematous reaction was recommended. Thus, the patient was given a plan of treatment that included four hundred milligrams of oral albendazole per day for three days, combined with topical steroids, emollients, and antihistamine tabs. Consequently, the lesions and itching resolved completely within two weeks (Fig. 2). The rapid response to the antihelminthic medication confirmed the initial diagnosis of CLM.

## DISCUSSION

Reports of atypical presentation and location of CLM have been increasing in recent years. The typical lesion is an erythematous, raised tunnel with a serpiginous arrangement of itchy papules and/or vesicles. However, the literature reported different types of unusual presentations such as folliculitis, bullous, eczematous, and ID reactions. Usually, the upper and lower parts of the extremities are the common sites, but other sites like buttocks, genitalia, chest, flanks, umbilicus, and webs of the fingers were also reported [8]. Hookwarm folliculitis is the most commonly reported type of atypical form of CLM. According to the available literature, the first case was reported more than 40 years ago, followed by more than 20 cases [5]. It is usually presented as multiple pruritic follicular papules, and pustules appear when many larvae infest the skin simultaneously. *Pelodera strongyloides* is a rare cause for the typical form; nevertheless, it is responsible for most CLM folliculitis cases. It prefers cold weather and usually affects the skin of cattle, dogs, horses, and sheep [1, 2, 5–7, 9]. Bullous type can occur, although the precise aetiology is unknown. However, some theories suggest that the separation of the epidermal cells occurs due to the secretion of larva lytic enzymes or hypersensitivity reaction to an unknown larval antigen [10–12]. Polymorphic Eczematous Dermatitis (PED) in the form of erythematous papules, plaque, pustules, excoriation, and hyperpigmentation with some serpiginous tracks was reported. It is localized to the back, as mentioned in this case and other published cases [13–16]. Alternatively, it could be widespread all over the body, which is more common in India, where hookworm infestations are common [17]. PED is nonspecific and difficult to diagnose and can mimic other dermatoses such as scabies, folliculitis, and contact dermatitis. Finally, ID reaction was reported in only one case since a palmoplantar eruption occurred after the patient received the treatment for HCLM [18].

Cellulitis is one of the complications of CLM, which is usually caused by *Streptococcus pyogenes*. Moreover, allergic reactions may occur, and Loeffler’s syndrome is sometimes reported [19]. Often, the diagnosis is made by history and physical examination. However, peripheral eosinophilia and elevated immunoglobulin E (IgE) levels are observed in certain cases. On the other hand, a skin biopsy is not required to diagnose CLM but may confirm the diagnosis by the appearance of the larvae or their tunnels within the epidermis or dermis. When observed under dermoscopy, the larvae’s body would appear as an oval structure with a brown centre and a yellow border [20, 21]. Immunodiagnostic methods can be helpful, such as detecting antigens from adult warm and Enzyme-Linked Immunosorbent Assay (ELISA) [22]. In addition, High-Frequency Ultrasound and Advanced Video-dermoscopy are new techniques used in diagnosing CLM [23]. Perhaps the treatment of CLM is easy; resistance to treatment and relapse are reported [24, 25]. Albendazole, thiabendazole, or ivermectin are the recommended systemic treatments. In addition, topical treatment with metronidazole, ivermectin, or thiabendazole creams may also be considered [12]. Application of topical permethrin 5% twice daily for two weeks is mentioned as an effective treatment [26, 27]. CLM presents with symptoms that are similar to other common skin conditions such as allergic contact dermatitis, urticarial factitis, and various other forms of dermatitis. Other possible diagnoses that share similar symptoms include pyodermas, subcutaneous nodules, granulomas caused by other species, and different types of myiasis [1]. CLM should also be differentiated from other creeping eruptions, such as Cutaneous Pilli Migrance, a rare form of skin disease in which a hair shaft migrates beneath the skin’s surface, leaving a linear erythema [28, 29]. Cercarial Dermatitis, Impetigo, Tinea Corporis, urticarial, photodermatitis, erythema chronicum migrans, cutaneous bacterial and fungal infections, photodermatitis and stings from Portuguese man-of-war or jellyfish and Scabies should be considered for differential diagnoses of CLM [2]. CLM can be differentiated from other conditions by its lack of serpentine migration. The most similar disease is larva currens, a migrating lesion caused by Strongyloides stercoralis. This disease progresses rapidly and erratically, with rates of several centimetres per hour and a haphazard pattern. It typically affects the perianal skin, thighs, or trunk. Fascioliasis, caused by the human trematode *Fasciola gigantica*, is another condition to consider. Non-infectious linear or serpiginous dermatoses such as jellyfish stings, lichenoid eruptions, and phytophotodermatitis are also non-migratory. Creeping ingrown hair is a rare possible diagnosis [3].

Diagnosis is typically made through clinical examination of the lesions, supported by the patient’s medical history. In some cases, the diagnosis may be missed due to its atypical appearance. Blood tests may reveal secondary infection, eosinophilia, and high serum IgE levels. Biopsies of the lesion are not recommended. Epiluminecent microscopy or ELISA are non-invasive methods of diagnosis [4].

## CONCLUSION

It is important to consider Cutaneous Larva Migrans (CLM) for any patient with an itchy skin rash that hasn’t improved with antihistamines or corticosteroids and has a recent travel history. CLM is a common disease that can present itself in unusual ways. Diagnosing Cutaneous Larva Migrans (CLM) holds great significance, especially in low-income tropical countries. The intense itching from CLM can cause open wounds, increasing susceptibility to bacterial infections. Untreated bacterial infections can progress to cellulitis, abscess formation, or sepsis, putting individuals’ health at risk. Moreover, the chronic nature of CLM, if left unaddressed, can diminish the quality of life for affected individuals, impacting sleep, social functioning and overall well-being.

## Data Availability

All the relevant study data is available from the corresponding author upon reasonable request.
